# The Penn Medical University: A Fatal Error

**Published:** 1881-09

**Authors:** Ad. Lippe

**Affiliations:** Philadelphia


### THE PENN MEDICAL UNIVERSITY: A FATAL ERROR.

AD. LIPPE, M. D., PHILADELPHIA.

IT is a fatal error to palm off the Penn Medical University as a homœopathic medical school. This fatal error has been committed by one who now pleads his own defense on pages 370 and 371 of THE HOMŒOPATHIC PHYSICIAN (Aug., 1881), over the signature of "The Historian."

The attention of the readers of this journal has already been called to the statement made by "The Historian" in the proceedings of The World's Homœopathic Convention of 1876 (page 801), that the Penn Medical University was, while it existed, a homœopathic institution. That such a statement constituted a fatal error, and that its publication was reprehensible, was clearly shown. "The Historian" asks for corrections, but fails to show that the Penn Medical University as a chartered medical school had any connection during its existence with any homœopathic association or school. On the contrary, it is evident that the said University was, to all intents and purposes, an *eclectic* medical school; that for that very reason some sort of homœopathy was taught, sandwiched in between several other sorts of treatment then in vogue. That a *kind* of homœopathy should be so taught in such a school was right and proper. But how can any one who has respect for the most ordinary logic, because a caricature of homœopathy was taught in this avowedly eclectic college, use that fact as an excuse for recording this school in history as a homœopathic institution?

"The Historian" enters the plea that some of the professors and students *became* homœopathists? Where, pray, should these eclectics have found shelter when their own schools were abandoned, but under the tree of "Freedom of Medical Opinion and Action," planted at Chicago, June 8th, 1870? The only person responsible for the publication of the assertion that the Penn Medical University was a homœopathic school is the *learned* gentleman who was intrusted by the American Institute of Homœopathy with the preparation of the "History of Homœopathy." Every member of the Institute, at all familiar with the *actual* history of homœopathy, is fully aware that this volume contains innumerable errors and *misstatements*—to use the mildest term. The acceptance of the volume by the Institute may have been construed by the compiler as a universal endorsement of everything it contains. If so, he commits a fatal error. When the seniors of the Institute, who were well aware of these departures from truth in the volume under discussion, neglected to expose them, and then and there accepted the volume without a protest, they committed a fatal error.

It was not expected that "The Historian" would appear again pleading his own cause, and now we must ask the question "what were his motives?" "The Historian" is the most interested party when he claims that the Penn Medical University was a homœopathic institute, and he now, by his persistent and ill-timed demand, that said University should go on record in history as one of the homœopathic institutions, opens a new field for discussion. This new "*Departure*" has more significance than appears on the surface, and would not have appeared as worth noticing if the "Historian" had not unnecessarily demanded a recognition which nobody ever thought of. This is not the place to dwell on the early history of the Eclectic Schools in this city, including the "Medical College of Pennsylvania," nor the various dodges by which the managers escaped the penalties of the law, nor to follow it up till at last public opinion, through the agency of the public press, brought down the most daring leader of that school, and placed him in durance vile to answer for his misdeeds. The question before us is a simple one—"*Was the Penn Medical University a Homœopathic Institute, and as such deserving to be mentioned in the History of Homœopathy?*" Our first inquiry is, what does constitute a homœopathic institution—a homœopathic college? Look at our charters and find the solution of the question. The charters expressively *command* that besides the collateral branches belonging to medical sciences, homœopathy should be taught; and why? because the allopathic schools not only neglected to accept the progresses made in therapeutics by homœopathy, but even refused to graduate homœopathic students. The charter of the late Penn Medical University contained no such imperative command to teach homœopathy, and that University never before claimed to be a homœopathic college. That University claimed to be, and was, an eclectic school—a school trying unsuccessfully to solve the problem that truth and error could co-exist. Quite another conclusion would be reached if we tamely accepted this Penn Medical University as a homœopathic institution. It would be this, that the positive command contained in *all* charters granted homœopathic colleges is not binding at all; that any college may teach just what the faculty pleases to teach, provided homœopathy is taught also, quite forgetful of the impropriety of teaching truth and error at the same time. If the American Institute, the representative association of our School, consents to incorporate *one* eclectic college among the homœopathic institutions, why not *all* of them, including Buchanan's school also? And if one or all eclectic colleges are accepted as homœopathic institutions, does such an acceptance not show clearly that the American Institute of Homœopathy knows of no difference between homœopathy and eclecticism? Was that the motive of "The Historian?" What other motive could he have? We, who follow the strict inductive method of Hahnemann, can find but this solution. Preposterous as such a proposition surely must appear, we find ourselves, much to our distaste, compelled, not only to expose this fallacy, this very fatal error, but to comment on it.

The tree of "Freedom of Medical Opinion and Action" has brought forth fruits, and they have been presented to the profession at large from time to time; each year brought fruits in more and more profusion. The text-book of our healing art, *The Organon*, has been neglected, and our materia medica has been caricatured into a physiologo-pathological picture book. The followers of Hahnemann's strict inductive method were frequently called to account, and a strict adherence to principles was called dogmatism. Hahnemann himself was traduced, and was called visionary and fanatical, his followers were charged with dishonesty. At last the dominant school, taking advantage of the open violation of strictly homœopathic principles by many bold practitioners, who frequently resorted, not only to palliative methods, contrary to our well-known therapeutic law, but who even resorted to larger, more poisonous, doses than were ordinarily prescribed by the common practitioner, charged the whole school with dishonesty, and made it appear that homœopathy, as taught by Hahnemann, has been forever rejected by his own followers. The last and bitterest fruit this tree has produced is now offered to the profession at large, and we are asked to declare homœopathy and eclecticism synonymes. Shall we? Assuredly not! Homœopathy, as taught by Hahnemann and as it stands progressively developed by the true healer, will never be perverted into eclecticism; let "The Historian" remember it!

